# Submicroscopic *Plasmodium* prevalence in relation to malaria incidence in 20 villages in western Cambodia

**DOI:** 10.1186/s12936-017-1703-5

**Published:** 2017-01-31

**Authors:** Rupam Tripura, Thomas J. Peto, Christianne C. Veugen, Chea Nguon, Chan Davoeung, Nicola James, Mehul Dhorda, Richard J. Maude, Jureeporn Duanguppama, Krittaya Patumrat, Mallika Imwong, Lorenz von Seidlein, Martin P. Grobusch, Nicholas J. White, Arjen M. Dondorp

**Affiliations:** 1 Mahidol Oxford Tropical Medicine Research Unit, Faculty of Tropical Medicine, Mahidol University, 420/6 Rajvithi Rd, Rajthevee, Bangkok, 10400 Thailand; 2 Center of Tropical Medicine and Travel Medicine, Department of Infectious Diseases, Division of Internal Medicine, Academic Medical Center, University of Amsterdam, Amsterdam, The Netherlands; 3 Centre for Tropical Medicine and Global Health, Nuffield Department of Clinical Medicine, University of Oxford, Oxford, UK; 4 National Center for Parasitology, Entomology and Malaria Control, Phnom Penh, Cambodia; 5 Provincial Health Department, Battambang, Cambodia; 6 Faculty of Public Health and Policy, London School of Hygiene and Tropical Medicine, London, UK; 7 World-Wide Antimalarial Resistance Network, Mahidol University, Bangkok, Thailand; 8 Department of Epidemiology, Harvard T. H. Chan School of Public Health, Harvard University, Boston, USA; 9 Department of Molecular Tropical Medicine and Genetics, Faculty of Tropical Medicine, Mahidol University, Bangkok, Thailand

**Keywords:** Malaria, *Plasmodium Falciparum*, *Plasmodium vivax*, Asymptomatic, Sub-clinical, Sub-microscopic, uPCR, Cambodia

## Abstract

**Background:**

Cambodia has seen a marked reduction in the incidence of *Plasmodium falciparum* over the past decade without a corresponding decline in *Plasmodium vivax* incidence. It is unknown to what extent local transmission is sustained by a chain of clinical and sub-clinical infections or by continued re-introduction via migration. Using an ultrasensitive molecular technique, 20 villages in western Cambodia were surveyed to detect the low season prevalence of *P. falciparum* and *P. vivax* and local treatment records were reviewed.

**Methods:**

During March to May 2015 cross-sectional surveys were conducted in 20 villages in Battambang, western Cambodia. Demographic and epidemiological data and venous blood samples were collected from 50 randomly selected adult volunteers in each village. Blood was tested for *Plasmodium* infections by rapid diagnostic test (RDT), microscopy and high volume (0.5 ml packed red blood cell) quantitative polymerase chain reaction (uPCR). Positive samples were analysed by nested PCR to determine the *Plasmodium* species. Malaria case records were collected from the Provincial Health Department and village malaria workers to determine incidence and migration status.

**Results:**

Among the 1000 participants, 91 (9.1%) were positive for any *Plasmodium* infection by uPCR, seven (0.7%) by microscopy, and two (0.2%) by RDT. uPCR *P. vivax* prevalence was 6.6%, *P. falciparum* 0.7%, and undetermined *Plasmodium* species 1.8%. Being male (adjusted OR 2.0; 95% CI 1.2-3.4); being a young adult <30 years (aOR 2.1; 95% CI 1.3–3.4); recent forest travel (aOR 2.8; 95% CI 1.6–4.8); and, a history of malaria (aOR 5.2; 95% CI 2.5–10.7) were independent risk factors for parasitaemia. Of the clinical malaria cases diagnosed by village malaria workers, 43.9% (297/634) and 38.4% (201/523) were among migrants in 2013 and in 2014, respectively. *Plasmodium vivax* prevalence determined by uPCR significantly correlated with vivax malaria incidences in both 2014 and 2015 (p = 0.001 and 0.002, respectively), whereas no relationship was observed in falciparum malaria (p = 0.36 and p = 0.59, respectively).

**Discussion:**

There was heterogeneity in the malaria parasite reservoir between villages, and *Plasmodium* prevalence correlated with subsequent malaria incidence. The association was attributable chiefly to *P. vivax* infections, which were nine-fold more prevalent than *P. falciparum* infections. In the absence of a radical cure with 8-aminoquinolines, *P. vivax* transmission will continue even as *P. falciparum* prevalence declines. Migration was associated with over a third of incident cases of clinical malaria.

*Trial registration* clinicaltrials.gov (NCT01872702). Registered 4 June 2013

**Electronic supplementary material:**

The online version of this article (doi:10.1186/s12936-017-1703-5) contains supplementary material, which is available to authorized users.

## Background

Malaria incidence in Cambodia has decreased by approximately 42% from 2009 to 2015 [1]. The treatment records of the National Malaria Control Programme, which do not include the private sector, report that this was driven chiefly by a decline of 55% in *Plasmodium falciparum*, whereas *Plasmodium vivax* declined by only 17%. This progress was due to the roll-out of rapid diagnostic tests (RDTs), treatment of confirmed malaria cases with artemisinin combination therapy (ACT) provided by village malaria workers (VMWs), and the distribution of insecticide treated bed-nets (ITNs) [1].

The Thai-Cambodian border area around Pailin Province in western Cambodia has been a cradle of anti-malarial drug resistance. In the 1960s, resistance of *P. falciparum* to chloroquine emerged from this area before spreading to Asia and Africa and causing millions of deaths [2]. Since 2007 several studies have shown that *P. falciparum* in western Cambodia has become resistant to artemisinins and more recently to the partner drugs used in ACT, such as piperaquine [3–6]. Subsequently, artemisinin-resistant *Plasmodium* parasites were found on the Thai-Myanmar border and other parts of Southeast Asia [7]. ACT offers the most effective anti-malarial drugs currently available, and the loss of these drugs would be a setback of potentially disastrous proportions for global malaria control and for elimination strategies in Southeast Asia [8, 9]. There is increasing evidence that in order to prevent the spread of artemisinin-resistant *P. falciparum* it is necessary to completely interrupt *P. falciparum* transmission [10–12].

Previous surveys have described an asymptomatic reservoir of malaria in Cambodia [13, 14]. Microscopy and RDTs are not sensitive enough to detect the majority of sub-clinical *Plasmodium* infections [13, 15, 16]. High-volume, ultra-sensitive, quantitative polymerase chain reaction (uPCR) is a recently developed method that can detect approximately 85% of all sub-clinical *Plasmodium* parasitaemias and thus characterize in detail the micro-epidemiology of malaria in areas of low transmission. These sub-clinical parasitaemias may play an important role in the transmission of infection, especially in areas of low and seasonal transmission [17, 18].

There are large seasonal population movements in and out of Samlout District in Battambang Province where the study was conducted. People from the district, predominantly men, spend time working outside the district and some return infected with malaria parasites. Agricultural labourers from other provinces come into the district to work during harvests and may harbour sub-clinical malaria infections acquired in other parts of the country. Seasonal work in forests collecting fruit, vines and hunting small game puts local people at risk of infection. These may contribute disproportionately to the transmission of malaria parasites. Battambang Provincial Health Department reports that approximately 83% of all malaria cases were aged 15–49 years, and 82% were males in 2014. The proportion of malaria cases among migrant workers in Battambang increased from 19% in 2009 to around 40% in 2014. In Cambodian border areas, the role of local, national and international migrants in malaria transmission has been acknowledged; however, the attributable fraction of infections has not been determined [19]. Outdoor and forest-acquired malaria infections continue to be important sources of transmission despite high rates of deforestation in the border areas of Cambodia.

Accurate malaria risk stratification is important for targeting control and elimination activities. Following the decline in *P. falciparum* infections over the past decade in western Cambodia, it is unknown to what extent local transmission is sustained by a chain of clinical infections, a reservoir of sub-microscopic malaria, or by continued introduction via migration. 20 villages in western Cambodia were surveyed to detect the sub-clinical prevalence of *Plasmodium* infections in an area where transmission is low but persistent and analysed the data with reference to available treatment records.

## Methods

### Study site

**Fig. 1:**
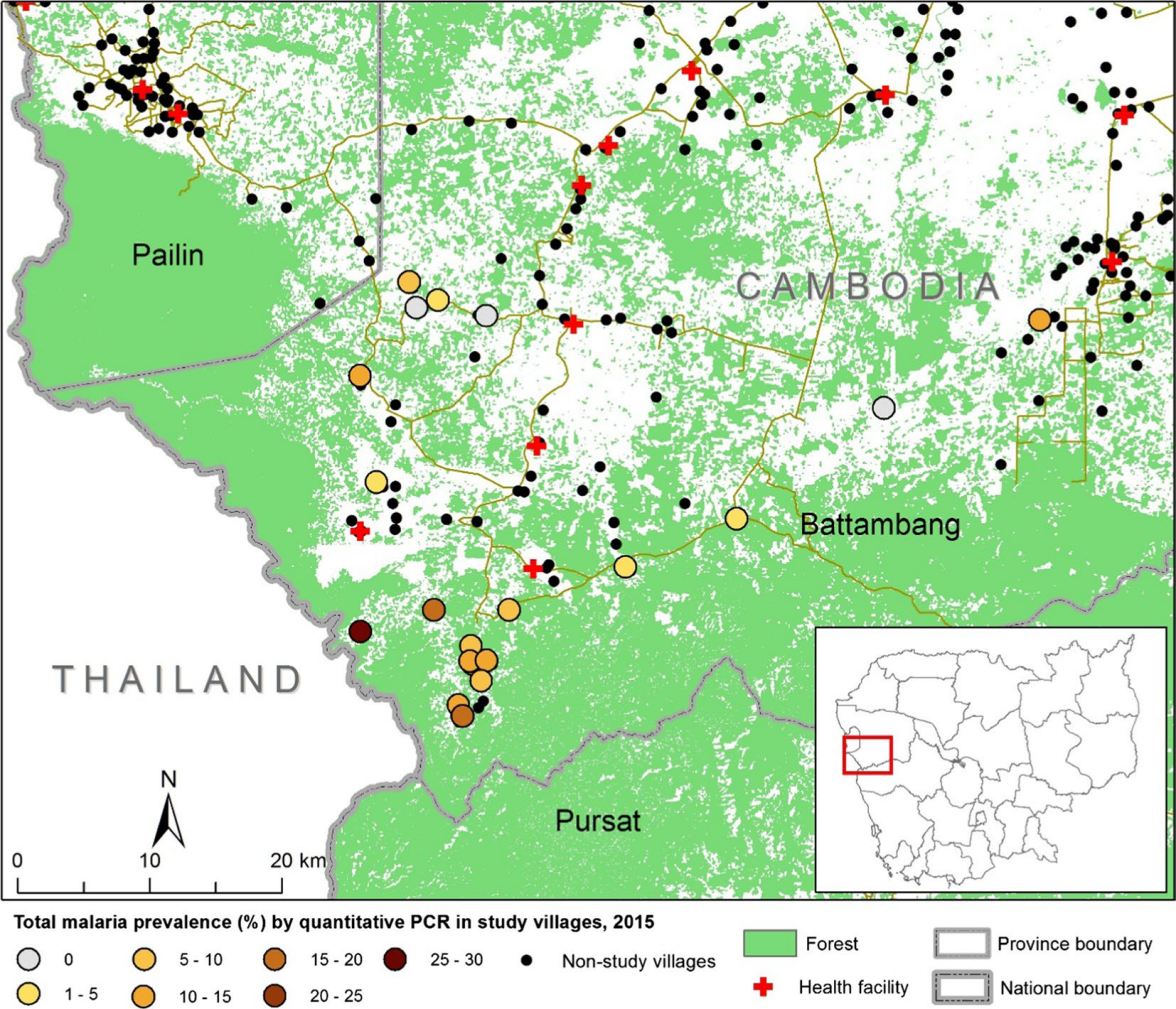
*Plasmodium* species determined by quantitative PCR in twenty villages in Battambang Province in 2015. Villages were selected for the survey according to their high malaria incidence in 2013–2014 from provincial malaria records; or, for villages with no village malaria worker, on the advice of local health centres which received referred malaria patients

During the low season of malaria in March to May 2015, cross-sectional surveys were conducted in 20 villages in Samlout District, Battambang Province, western Cambodia. All available VMW records and provincial malaria data were reviewed. Samlout District lies to the south of Pailin Province, north of Pursat Province and the Cardamom Mountains, and east of Thailand (Fig. 1). Samlout is primarily an agricultural district with forested areas along the border. Samlout was chosen for this study as it has villages with some of the highest incidence of clinical malaria in Battambang Province and seasonal transmission.

### Selection of villages and participants

Within Samlout District, 18 villages with the highest malaria incidence in 2014 were selected along with two neighbouring villages, totalling 20. A census based on pre-existing village population lists was updated and household lists were prepared. Surveyed households were selected using randomization methods from the list to ensure that every household had an equal chance of being selected. A stratified sampling method obtained approximately equal samples of adults aged 18 years or over from four groups (male/female, under/over 30 years). Unless the total number of households in a village was fewer than 50, no more than one person per household was selected. For the villages with fewer than 50 households, more than one participant was randomly selected from the list. In cases of refusal or a person being absent on the survey day, replacement participants were selected randomly from the same household or the next household on the list. Pregnant women were excluded from the randomization as they would be ineligible to join a mass anti-malarial treatment study which followed this survey.

### Community engagement and informed consent

Public meetings were conducted in Khmer by a dedicated Community Engagement Team (CE-Team) to inform the commune authorities, VMWs and village leaders about the study. All residents were invited to a meeting prior to the survey where the consent form was read aloud and questions were answered. Individual written informed consent was obtained immediately prior to sample collection and interview. A fingerprint was obtained from participants who could not read and write and a witness countersigned their consent form.

### Survey methods

Anthropometric and demographic data were collected, including tympanic temperature, height, weight, health status, malaria history, bed net use, travel history, and occupation; 1.5 ml of venous blood was collected in an EDTA tube. Samples were kept in a cool box and sent to a nearby hospital laboratory on the same day. If a participant was febrile (defined as tympanic temperature ≥37.5 C°) an RDT (SD BIOLINE Malaria Ag P.f/P.v, Standard Diagnostics, Inc, Gyeonggi-do, Republic of Korea) was performed immediately, and if positive, treatment was provided by the VMW according to national guidelines.

### Laboratory methods

The samples were separated; 200 µl of whole blood was used to prepare a dried blood spot on filter paper, a blood smear for microscopy, and to perform haemoglobin assay (HemoCue^®^ Hb 201^+^Analyzer SE-262 71 Angeltholm, Sweden). Blood was tested for malaria by RDT and microscopy (asexual parasites/500 white blood cells (WBCs)). The remaining blood was centrifuged and plasma, buffy coat and 500 µl of packed red blood cells (pRBCs) for uPCR were separated into three different cryo-tubes and stored at −80 °C. Ten per cent negative controls of total participant samples were added for each village. All the samples were transported to the Mahidol University Faculty of Tropical Medicine molecular laboratory in Bangkok.

### Molecular laboratory procedures

A detailed description, evaluation and validation of the high-volume ultrasensitive (uPCR) methodology has been reported previously [15]. In brief, batches of 500 µl of pRBCs were thawed and the DNA template, for detection and quantification of *Plasmodium* by PCR, was extracted and purified. An ultrasensitive quantitative real-time PCR method was used to detect the presence of malaria parasites and to estimate the numbers (genomes) of parasite per millilitre from each sample. The 18S rRNA-targeting primers and hydrolysis probes used in the assay have been validated and are highly specific for *Plasmodium* species. The lower limit of accurate quantitation of this method is 22 parasites/ml of whole blood [15].

A Quanti-Tect Multiplex PCR No ROX^®^ (QIAGEN, Hilden, Germany) was used, and the PCR mixture and the cycling conditions were as per manufacturer’s instructions. For samples containing parasite DNA by uPCR analysis, *Plasmodium* species detection was attempted using real time PCR protocols specific to 18sRNA of *P. falciparum* and *P. vivax*. Samples for which *Plasmodium* species were not determined were reported as ‘unknown species’.

To detect polymorphisms associated with reduced susceptibility to artemisinin derivatives the open-reading frame of the PF3D7_1343700 kelch propeller domain was amplified using a nested PCR protocol [7]. Purified PCR products were sequenced at Macrogen, Republic of Korea and analysed using BioEdit version 7.1.3.0., using the 3D7 kelch13 sequence as reference (Accession: XM_001350122.1).

### Statistical analysis and data management

Data were collected on case record forms and entered on smartphones before being exported into Open Clinica. Survey data were analysed by univariate and multivariate methods to identify risk factors for parasitaemia. Odds ratios with 95% confidence intervals were calculated to explore which variables were independently associated with subclinical infection. The Pearson Chi squared test was used to test for the significance of associations. Variables that were independently associated with sub-clinical parasitaemia at the level of p < 0.05 were explored by logistic regression to produce adjusted odds ratios. Incidence rate was calculated by dividing total malaria cases in a given year with the total village population and multiplied by 1,000. Malaria incidence and prevalence were compared by linear regression. The analysis was done in STATA 14.0 (Timberlake, USA).

### Additional data sources and clinical malaria incidence

Malaria case reports were obtained from the Provincial Health Department and VMW records. Available treatment records were reviewed by cross-checking with the VMWs to confirm the identity of patients. The incidence of clinical malaria for 2014 and 2015 was compared to the prevalence of malaria parasites detected by uPCR in March and April 2015 for each village. Villages with no Health Department records were excluded from this comparison. For villages where only a hamlet (Chakriya: CKA, Samlaut: SLT, Kompoung Tuk A: KTA and Kompoung Tuk B: KTB) was selected for the prevalence survey, the incidence rate of the whole village population was used for the analyses. All prevalence survey participants were from registered households and were considered to be residents.

## Results

### Description of the study population

Population size and malaria incidence in 2014-2015 differed significantly among the 20 study villages. Population size ranged from 147 (in Ou Nounong: ONG) to 1012 (in Ouda: ODA). Malaria incidence per thousand ranged between two and 224 among villages in 2014 and 2-166 in 2015. Two villages (CKA, Miko: MKO) in 2014 and two villages (CKA, Peam: PEM) in 2015 were not listed in Provincial Health Department records, so no incidence data were available for these villages (Table 1 **)**. The median age of the study participants was 33 years (range from 18 to 78 years). The median age of male and female participants were 34 years (18–72 years) and 32.5 years (17–78 years), respectively; 11.7% (117/1000) subjects had a temperature of 37.5 °C or higher; 65.4% (654/1000) had a history of malaria at any time prior to the survey.

**Table 1:** *Plasmodium* species prevalence by uPCR in 2015 cross-sectional surveys and malaria incidence from 2014-15

Village code	uPCR prevalence				Clinical cases from health records			
	*P. falciparum* n/50 (%)	*P. vivax* n/50 (%)	^*a*^ *P. species* n/50 (%)	Population 2014–2015	2014 PF or mix cases (incidence per 1000)	2014 All malaria cases (incidence per 1000)	2015 Pf or mix cases (incidence per 1000)	2015 All malaria cases (incidence per 1000)
CKA	0	13 (26)	2 (4)	409	No record	No record	No record	No record
ONG	0	4 (8)	5 (10)	147	7 (47.6)	33 (224.5)	2 (13.6)	12 (81.6)
PTA	1 (2)	5 (10)	2 (4)	283	7 (24.7)	39 (137.8)	0 (0.0)	2 (7.1)
PRY	0	8 (16)	0	614	11 (17.9)	115 (187.3)	44 (71.7)	102 (166.1)
OTG	0	5 (10)	2 (4)	239	10 (41.8)	43 (179.9)	11 (46.0)	26 (108.8)
TTK	0	7 (14)	0	214	1 (4.7)	21 (98.1)	3 (14.0)	7 (32.7)
SLT	0	5 (10)	1 (2)	657	11 (16.7)	43 (65.4)	38 (57.8)	61 (92.8)
CMN	0	6 (12)	0	642	8 (12.5)	17 (26.5)	7 (10.9)	13 (20.2)
PEM	0	4 (8)	2 (4)	257	0.0	1 (3.9)	No record	No record
OTT	2 (4)	1 (2)	2 (4)	300	0.0	1 (3.3)	2 (6.7)	2 (6.7)
ODA	3 (6)	1 (2)	0	1012	28 (27.7)	35 (34.6)	19 (18.8)	22 (21.7)
OKH	0	2 (4)	1 (2)	631	3 (4.8)	45 (71.3)	15 (23.8)	29 (46.0)
VRM	1 (2)	2 (4)	0	291	28 (96.2)	71 (244.0)	8 (27.5)	28 (96.2)
KTB	0	1 (2)	0	748	5 (6.7)	10 (13.4)	1 (1.3)	3 (4.0)
SRH	0	1 (2)	0	562	3 (5.3)	6 (10.7)	2 (3.6)	3 (5.3)
MKO	0	0	1 (2)	313	No record	No record	3 (9.6)	15 (47.9)
OCL	0	1 (2)	0	456	0 (0.0)	1 (2.2)	3 (6.6)	5 (11.0)
CLK	0	0	0	275	3 (10.9)	6 (21.8)	2 (7.3)	3 (10.9)
KTA	0	0	0	748	5 (6.7)	10 (13.4)	1 (1.3)	3 (4.0)
APP	0	0	0	397	0 (0.0)	4 (10.1)	0 (0.0)	1 (2.5)

*Incidence* total malaria cases/total village population* 1000

*Prevalence* total *Plasmodium* species detected by uPCR/total number of test* 100

^*a*^Positive for *Plasmodium* by high-volume qPCR, but the parasite genome count was too low for subsequent nested PCR to determine *Plasmodium* species

### Prevalence of *Plasmodium species* by detection method

Among 1000 participants, 91 (9.1%) were positive for any *Plasmodium species* by uPCR, seven (0.7%) by microscopy, and two (0.2%) by RDT. By uPCR, *P. vivax* prevalence was 6.6% (66/1000), *P. falciparum* 0.7% (7/1000), and 1.8% (18/1000) had undetermined *Plasmodium* species. In Table 1 the parasite prevalence by uPCR is shown for each village.

The prevalence in the villages ranged from 0 to 30% by uPCR (test for heterogeneity p < 0.001)*. Plasmodium falciparum* parasites were detected by uPCR in four of the 20 villages, with *P. falciparum* prevalence ranging from 2 to 6% (p = 0.010). *Plasmodium vivax* infections were detected by uPCR in 16 villages, with *P. vivax* prevalence ranging between 2 and 26%, (p < 0.0001) and the undetermined *Plasmodium* species were detected in nine villages, prevalence ranging from 2 to 10% (p = 0.011 for heterogeneity). Three out of 20 villages with had no parasite prevalence by uPCR.

### Factors associated with *Plasmodium species* positivity by uPCR

Factors associated with any positive *Plasmodium* species by uPCR are shown in Table 2. *Plasmodium* species prevalence among males was 14.1% (66/402) versus 4.7% (25/532) among females p < 0.001; 11.5% (51/443) among those aged 18-29 versus 7.2% (40/557) among those aged 30 or above p = 0.018. *Plasmodium* prevalence was 6.8% (8/117) among those with temperature of 37.5 °C or higher versus 9.3% (83/883) among those with a temperature below 37.5 °C (p = 0.365). Among participants with previous history of clinical malaria, 12.5% (82/654) were parasitaemic versus 2.6% (9/346) among those who said they had never had malaria (p < 0.0001).

**Table 2:** Univariate associations between factors investigated for prevalence of *Plasmodium* infection by uPCR

Variable	Number	uPCR positive for *Plasmodium*	uPCR negative for *Plasmodium*	*p* value*	Odds ratio^a^	Adjusted odds ratio^b^
		Number (%)	Number (%)		(95% CI)	(95% CI)
Number	1000	91 (9)	909 (91)			
Gender				<0.0001	3.3 (2.0–5.4)	2.0 (1.2–3.4)
Male	468	66 (14)	402 (86)			
Female	532	25 (5)	507 (95)			
Age groups (years)				0.018	1.6 (1.1–2.6)	2.1 (1.3–3.4)
18–30	443	51 (11.5)	392 (88.5)			
>30	557	40 (7.2)	517 (92.8)			
Tympanic temperature (°C)				0.365	0.7 (0.3–1.5)	0.9 (0.4–1.9)
≤37.5	883	83 (9)	800 (91)			
≥37.5	117	8 (7)	109 (93)			
Self-reported fever in previous 48 h				0.255	0.6 (0.2–1.5)	0.7 (0.3–1.8)
Fever	87	5 (6)	82 (94)			
No fever	913	86 (9)	827 (91)			
Self-reported illness in the previous 48 h				0.121	0.7 (0.5–1.1)	0.9 (0.6–1.5)
Illness	506	39 (8)	467 (92)			
No illness	494	52 (11)	442 (89)			
Self-reported history of ever having had malaria in past				<0.0001	5.3 (2.6–10.9)	5.1 (2.5–10.7)
Yes	654	82 (13)	572 (87)			
No	346	9 (3)	337 (97)			
Forest visit in the previous 3 months				<0.0001	4.1 (2.5–6.5)	2.7 (1.6–4.8)
Yes	156	35 (22)	121 (78)			
No	844	56 (7)	788 (93)			
Travel in the previous 3 months				0.489	1.2 (0.7–1.9)	1.1 (0.7–1.8)
Yes	245	25 (10)	220 (90)			
No	755	66 (9)	689 (91)			
Bed net use				0.004	0.5 (0.3–0.8)	0.8 (0.5–1.5)
Everyday	849	68 (8)	781 (92)			
Sometimes or never	151	23 (15)	128 (85)			

* Pearson Chi Square test

^a^Mantel Haenszel odds

^b^Logistic regression, adjustment for gender, age group, temperature ≤ 37.5 °C

Prevalence of *Plasmodium* parasites among participants spending one or more nights in a forest within the same period was 22.4% (35/156) versus 6.6% (56/844) among those without any forest travel (p < 0.001). Among participants with parasitaemia, 27/91 (29.7%) had recently stayed overnight in the forest in the same province, and 8/91 (8.8%) had stayed in a forest in a different province; 56/91 (62%) did not have a history of overnight stay in the forest (p < 0.001). Parasite prevalence among participants with a history of travel outside the village within the previous 3 months was 10.2% (25/245) versus 8.7% (66/755) among those without any travel history (p = 0.489). Among those with parasites, 66.7% (66/91) did not travel in comparison to 27.3% (25/91) who travelled outside the village. Among the 25 positives who travelled outside the village, 44% (11/25) had stayed overnight in the forest.

Prevalence of parasitaemia was 8.0% (68/849) among those who used ITNs every night, 15.2% (20/132) among those who sometimes used ITNs, and 15.8% (3/19) among those who never used ITNs, p = 0.004. Parasite prevalence varied between the strata used for sampling: 19.9% (39/196) among young men, 9.9% (27/272) among older men, 4.8% (12/247) among young women, and 4.6% (13/285) among older women. Men were more likely to use ITNs irregularly or never (27.1%; 127/468) versus women (24.5%; 4/532) p < 0.0001. Men were also more likely than women to stay overnight in forests 28.4% (133/468) versus 4.3% (23/532), p < 0.0001. Young men (35.2%; 69/196) used ITNs irregularly or never, and 36.7% (72/196) had spent a night in a forest within the past 3 months.

After adjustment, being male (OR 2.0; 95% CI 1.2–3.5), younger adults (age 18–30; OR 2.1; 95% CI 1.3–3.4), recent forest travel (OR 2.6; 95% CI 1.5-4.5), and a prior history of malaria (OR 5.3; 95% CI 2.6–11.0) remained significantly associated with parasite prevalence.

**Fig. 2:**
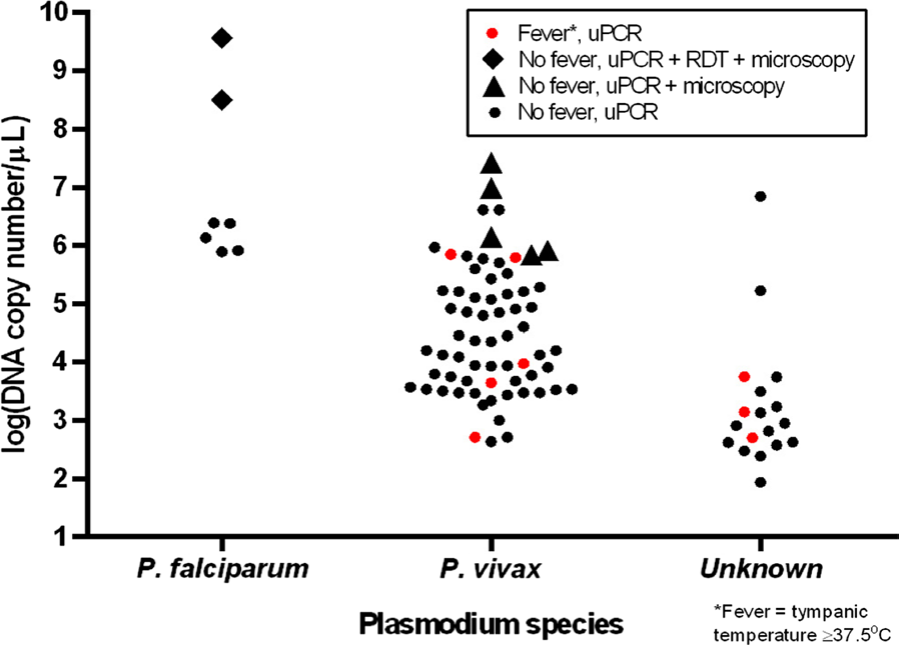
Parasite genome count by *Plasmodium* species, tympanic temperature ≥ 37.5 °C and method of detection

Parasitaemia was higher among *P. falciparum*-positive individuals than those with *P. vivax*. Parasitaemia was lowest among people with undetermined *Plasmodium* species where the species were not determined. Tympanic temperature was not associated with the level of parasitaemia for any *Plasmodium* species (Fig. 2).

### Sub-clinical *Plasmodium falciparum*

Among participants with *Plasmodium* parasitaemia, seven/91 had *P. falciparum* parasites. Among the seven with *P. falciparum* parasites, ages ranged from 22 to 49 years. None reported fever within the 48 h before screening and none had a temperature >37.5 °C. All reported having clinical malaria in the past, and five reported illness in the previous 48 h. Three came from one village, ODA, and two from another, Ou Ta Teak (OTK). Four had stayed in a local forest overnight in the previous 3 months, of whom two reported travel outside the village in the same period. Two were positive both by RDT and by microscopy. Only one of seven falciparum positives had a history of travel to another province within the previous 3 months. Five reported everyday ITN use.

### Markers of artemisinin resistance

Six of seven *P. falciparum* parasite strains detected by uPCR could be sequenced for *PfKelch* 13 mutations. All six *P. falciparum* isolates obtained *Pfkelch13* C580Y mutation.

### Association between sub-clinical prevalence and clinical malaria incidence

**Fig. 3:**
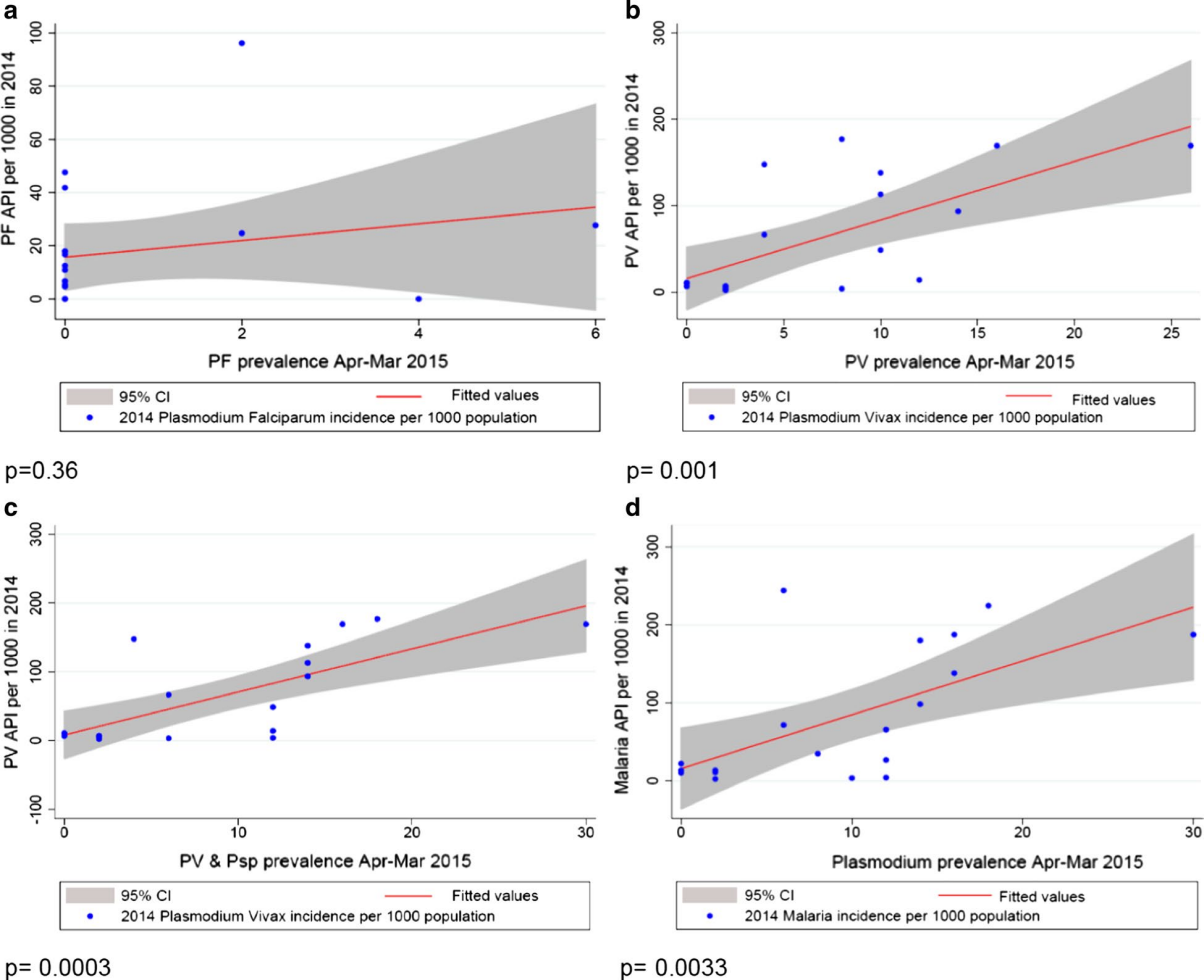
*Plasmodium* species prevalence by uPCR in 2015 and incidence of malaria cases in 2014. **a** *Plasmodium falciparum* prevalence by uPCR and falciparum malaria and mixed malaria incidence 2014; **b** *Plasmodium vivax* prevalence by uPCR and vivax malaria incidence 2014; **c** *Plasmodium vivax* and undetermined *Plasmodium* species prevalence by uPCR and vivax malaria incidence 2014; **d** *Plasmodium* prevalence by uPCR and malaria incidence 2014. In each graph, X axis represents *Plasmodium* prevalence by uPCR during March–April 2015 and Y axis represents malaria cases per 1000 population in a 1-year period. *API* annual parasite incidence. API = (confirmed cases during 1 year/population under surveillance) ×1000. In 2014, *19 out of 20 villages malaria incidence rates were available from Battambang Provincial Health Department and are included in the analyses, except MKO

**Fig. 4:**
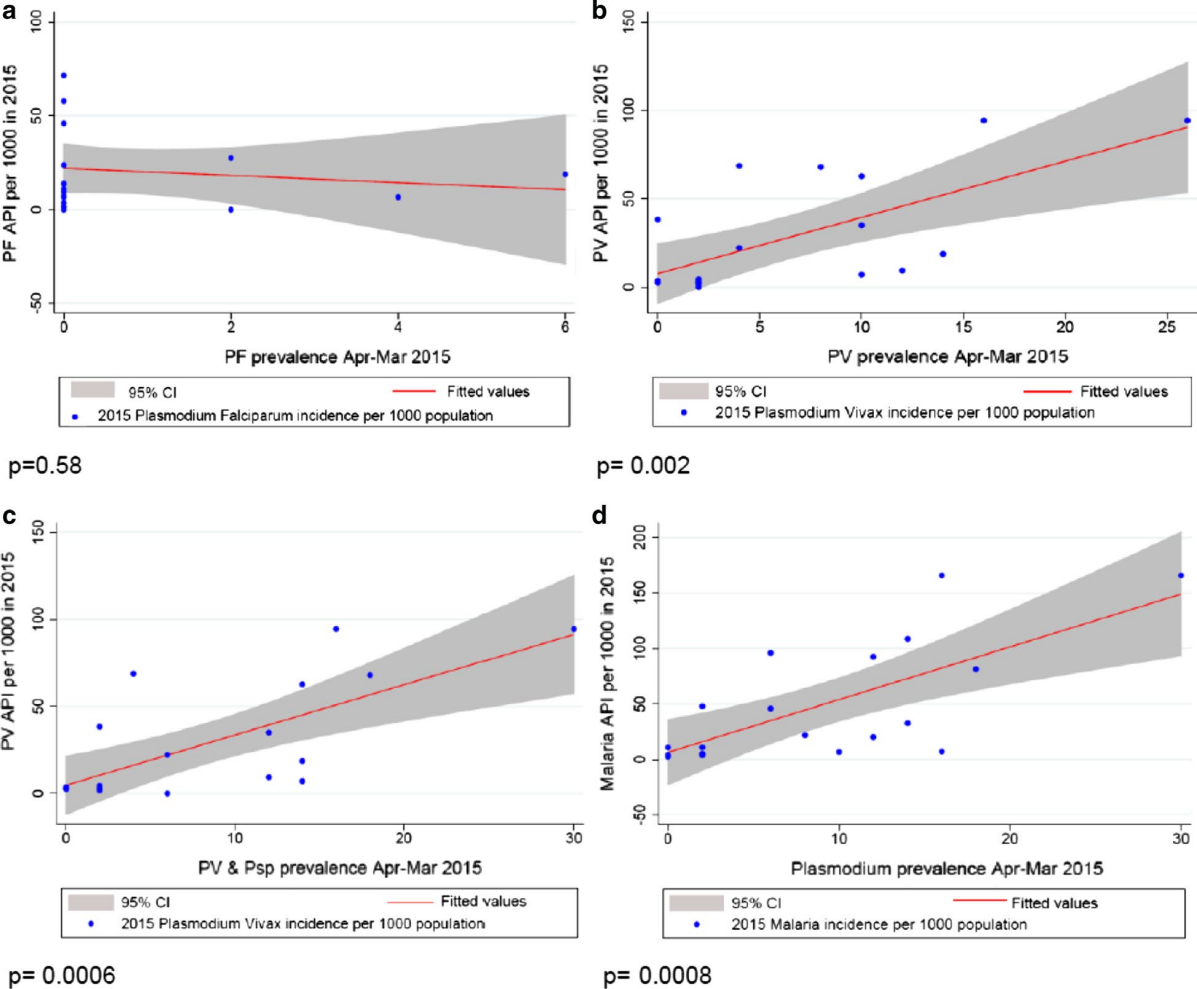
*Plasmodium* species prevalence by uPCR and incidence of malaria cases in 2015. **a** *Plasmodium falciparum* prevalence by uPCR and falciparum or mixed malaria incidence 2015; **b** *Plasmodium vivax* prevalence by uPCR and vivax Malaria incidence 2015; **c** *Plasmodium vivax* and undetermined *Plasmodium* species prevalence by uPCR and vivax malaria incidence 2015; **d** *Plasmodium* prevalence by uPCR and malaria incidence 2015. In each graph, X axis represents *Plasmodium* prevalence by uPCR during March–April 2015 and Y axis represents malaria cases per 1000 population in a 1-year period, *API* annual parasite incidence. API = (confirmed cases during 1 year/population under surveillance) ×1000. In 2015, *19 out of 20 villages malaria incidence rates were available from Battambang Provincial Health Department and are included in the analyses, except for PEM

Malaria incidence was obtained for 19/20 survey locations from provincial health department records in 2014 and 2015. Overall malaria prevalence in the March–April 2015 survey measured by uPCR significantly correlated with the incidence in each of 2014 and 2015 (p = 0.003 and p < 0.001), respectively. *Plasmodium vivax* prevalence correlated with *P. vivax* incidence (p = 0.001 and p = 0.002), respectively. No correlation was observed between *P. falciparum* prevalence and incidence (p = 0.36 and p = 0.59). The combined prevalence of *P. vivax* and undetermined *Plasmodium* parasites (p < 0.001) was also correlated with *P. vivax* incidence. This was done as previous surveys found approximately 90% of undetermined *Plasmodium* species detected by uPCR in western Cambodia were later identified as *P. vivax* during 12 months of prospective follow-up (Additional file 1: Figure S1) [14] (Figs. 3, 4).

### Village malaria worker treatment records

**Fig. 5:**
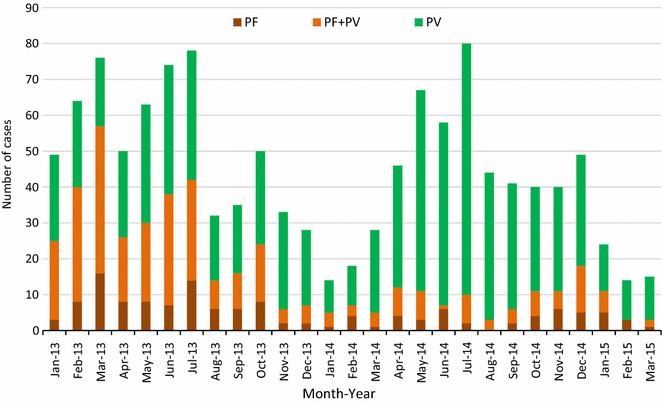
Clinical malaria cases in the survey villages recorded by village malaria workers from 2013 to March 2015. *Plasmodium falciparum* mono-infections are *red*, mixed *P. falciparum* and *P. vivax* are *orange*, and *P. vivax* mono-infections are *green*

Among all clinical malaria cases reported by VMWs, 154 (12.7%) were among people aged 45 years and above, 842 (69.6%) aged 15–45, 189 (15.6%) aged five to 15, and (2.1%) aged <5 years. People recorded as migrants (no permanent address in the village) accounted for (297/634) 43.9% of all malaria cases in 2013, and (201/523) 38.4% in 2014; 914/1,210 (75.5%) of subjects were male and 295/1,210 (24.5%) were female. There was an approximately three-fold reduction in the number of *P. falciparum* and mixed *P. falciparum* and *P. vivax* cases from 88 (13.9%) and 237 (37.4%) in 2013, to 36 (7.3%) and 66 (13.0%) in 2014, respectively. The number and proportion of *P. vivax* cases increased from 309 (48.7%) in 2013 to 417 (79.7%) in 2014 respectively (Fig. 5).

## Discussion

### Spatial heterogeneity and risk factors for parasitaemia

Variations observed in the prevalence of malaria in the study villages were consistent with a recent study in the northeast of Cambodia, which also found evidence of heterogeneity of malaria prevalence in nearby villages.

In the low season nearly one in ten people had malaria parasites. Sub-clinical vivax parasitaemia had a higher prevalence than sub-clinical falciparum parasitaemia. The predominance of *P. vivax* in the infections detected in the survey is consistent with local treatment records. A significant proportion of clinical falciparum cases were among migrants. More detailed information on travel history and places of overnight stay in malaria-endemic areas would be helpful to better explain where malaria infections were acquired.

Young men were at greater risk of clinical malaria and had a higher prevalence of sub-microscopic malaria parasites compared to older men or to adult women. Young men were also more likely to spend time in forests and use ITNs irregularly or not at all, consistent with other studies in Cambodia [20, 21].

*Plasmodium vivax* prevalence determined by uPCR significantly correlated with vivax malaria incidence in each of 2014 and 2015, whereas no relationship was observed for *P. falciparum*. Due to the shorter duration of *P. falciparum* infections, it is unlikely that a single snapshot of *P. falciparum* by uPCR during the dry season would be able to describe the picture of *P. falciparum* infections throughout the whole year. Whereas for *P. vivax*, the higher persistence of sub-clinical infections means a survey in the dry season reveals a large reservoir of infections. In a previous longitudinal study a large proportion of sub-clinical *P. vivax* persisted through the year and seasonality had little effect on the number of detectable infections [14]. Even with testing a relatively small sample of each village’s population, there was a significant association between numbers with clinical and sub-clinical *P. vivax*.

### Implications for vivax treatment

There was a significant association between a prior history of malaria and having vivax parasitaemia. These self-reported histories (which often dated back many years) are not a promising target to guide screening or treatment as the absolute risk difference was 3% (12 versus 9% overall) and two-thirds of all participants reported a history of clinical malaria. However, clinical treatment records of confirmed malaria diagnoses are a more reliable predictor of persistent vivax parasitaemia [22].

### Implications for falciparum elimination

To accelerate malaria elimination, malaria control programmes in Cambodia and other countries are evaluating various strategies for mass drug administration (MDA) [11, 23–25]. Although in the dry season *P. falciparum* prevalence in these villages was very low, six of seven uPCR-positive *P. falciparum* infections did not have recent history of travel to another province, suggesting some ongoing local transmission. Without the contribution of migrants to the clinical malaria records Samlout District may be close to interruption of *P. falciparum* transmission, but imported cases may undermine elimination. In a recent study in neighbouring Pailin Province, a high proportion of *P. falciparum* clinical cases were found to have C580Y Kelch mutations, and in 2013-14 prevalence surveys in Pailin, approximately half of all asymptomatic *P. falciparum* infections had markers associated with artemisinin resistance [7, 14]. In Laos, a high proportion of asymptomatic parasitaemias had the same marker associated with artemisinin resistance [26].

### Limitations and strengths

The cross-sectional survey design was unable to resolve whether past clinical malaria left persistent parasites or whether these individuals were parasitaemic because they were generally at a higher risk. This study was conducted during the dry season and studies from Pailin in 2013–2014 reported a several-fold variation in *P. falciparum* prevalence over one year; therefore, it is not possible to estimate the overall contribution of these infections to transmission in these communities. It was also not possible to determine the *Plasmodium* species of some parasitaemias. Generally these were the participants with the lowest quantity of parasite DNA. The incidence data could be incomplete as VMW data from 2014 may have missed some people who went to other places for diagnosis and treatment.

## Conclusions

The low season reservoir consisted chiefly of *P. vivax* infections with very low *P. falciparum* prevalence. *Plasmodium vivax* prevalence correlated with incidence of clinical malaria episodes while *P. falciparum* did not. Knowledge of the crude *P. falciparum* incidence alone was not a reliable tool to predict the hidden parasite reservoir during the dry season in this instance. More detailed knowledge of the characteristics of the *P. falciparum* cases, such as occupation and travel history could potentially distinguish indigenous and imported malaria and thereby improve the use of local treatment records to predict the sub-clinical *P. falciparum* reservoir. This can be a particularly useful tool to target malaria elimination activities such as mass screening and treatment or mass anti-malarial drug administration.

Much clinical *P. falciparum* was among migrants, forest workers or those recently returned from other provinces, suggesting transmission inside the villages themselves is now very low. Young men were more likely to have sub-microscopic malaria than older men or females of any age and to exhibit behaviour that exposes them to the risk of infection (and transmission). Imported malaria has the potential to undermine provincial *P. falciparum* elimination efforts as clinical and sub-clinical infections are brought into the district by migrant workers and by local people returning from other provinces.

The spatial heterogeneity of *Plasmodium* prevalence between neighbouring villages shows that there is important variability in malaria epidemiology at a local level. Knowledge of the recent, detailed travel history of clinical malaria cases could provide crucial information about the probable source of infection and could help determine the risk of infection within individual villages. Even in villages with indigenous transmission, strategies to screen or treat sub-microscopic malaria in the dry season may be undermined by subsequent immigration of parasitaemic individuals. Therefore, targeting high-risk groups, e.g., migrant labourers, forest goers, by offering free screening and treatment, may prevent re-importation of malaria parasites from areas of higher transmission to areas in pre-elimination state and this could be an effective tool to eliminate falciparum malaria. Simultaneously, mass treatment in high transmission areas around the start of the malaria transmission season at community level may reduce the incidence of falciparum malaria significantly. It could have a collateral effect on a whole geographical area moving towards malaria elimination.

## Additional file

**Additional file 1: Figure S1.** Malaria prevalence by uPCR and incidence per 1000 in 2015 in 16 villages.

## Abbreviations

ACT: artemisinin-based combination therapy
DHA-PQP: dihydroartemisinin–piperaquine phosphate
EDTA: ethylenediaminetetraacetic acid
ITNs: insecticide-treated bed nets
MDA: mass drug administration
nested PCR: nested polymerase chain reaction
uPCR: ultrasensitive polymerase chain reaction
VMW_s_: village malaria workers
