# Supplementary material for: Submicroscopic Plasmodium prevalence in relation to malaria incidence in 20 villages in western Cambodia
Source: Malar J. 2017 Jan 31;16:56. doi: 10.1186/s12936-017-1703-5 (PMC5282880; doi:10.1186/s12936-017-1703-5)
Supplement: Supplementary file 1 [file 12936_2017_1703_MOESM1_ESM.docx]

a.

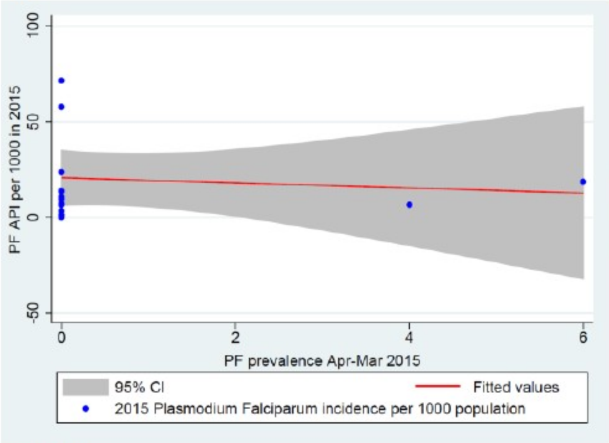

p=0.7306

b.

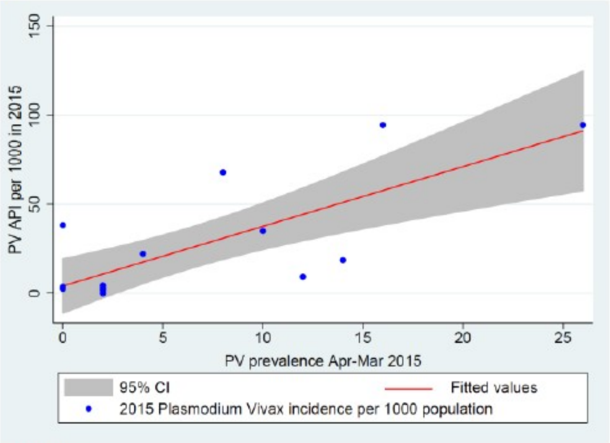

p=0.0005

c

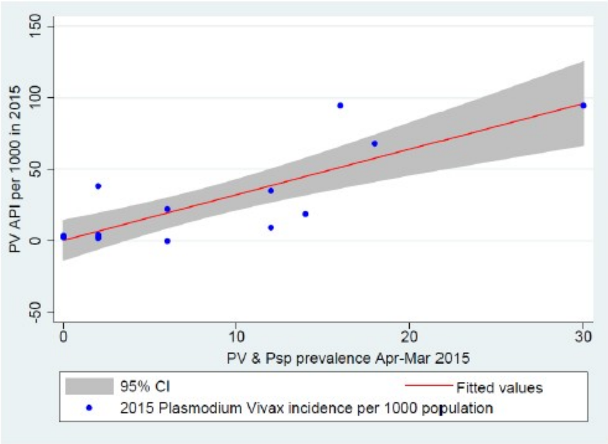

p=0.0001

d

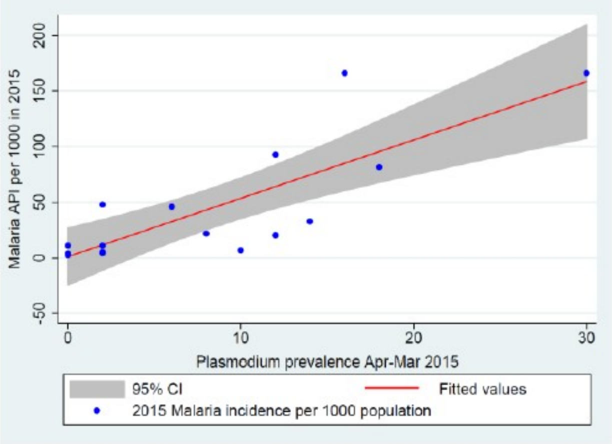

p= 0.0002
